# Skeletal Muscle Transcriptomic Responses to a High-Fat, High-Sugar Diet Across Sex and Maturation in Mice

**DOI:** 10.3390/nu18203354

**Published:** 2026-10-13

**Authors:** Emiliana Giacomello, Pablo Giraudi, Natalia Rosso, Silvia Gazzin, Claudio Tiribelli, Luana Toniolo

**Affiliations:** 1 Department of Medicine, Surgery and Health Sciences, University of Trieste, 34149 Trieste, Italy; 2 Fondazione Italiana Fegato MASLD Unit and Brain-Liver Unit “Rita Moretti”-Bldg. Q, AREA Science Park, ss14, Km 163.5, Basovizza, 34149 Trieste, Italy; pablo.giraudi@fegato.it (P.G.); natalia.rosso@fegato.it (N.R.); silvia.gazzin@fegato.it (S.G.); ctliver@fegato.it (C.T.); 3 Laboratory of Muscle Biophysics, Department of Biomedical Sciences, University of Padova, 35131 Padova, Italy

**Keywords:** obesity, skeletal muscle, high-fat high-sugar diet, sexual dimorphism, transcriptomics

## Abstract

Background/Objective: Pediatric obesity represents a paramount problem for global health that affects children’s actual quality of life, dramatically compromising their future health. Obesity has been demonstrated to alter skeletal muscle, a highly plastic tissue with dimorphic characteristics, which changes during development, homeostasis, adaptive responses, and diseases. Since it is responsible for 80% of insulin-stimulated glucose uptake, its alteration establishes a vicious circle profoundly impacting overall metabolism. Although obesogenic diets are known to distinctly affect adult male and female muscles, scarce information is available on growing individuals. Therefore, we performed an exploratory analysis on the early effects of an obesogenic diet in murine muscles during maturation. Methods: We analyzed the transcriptome of tibialis anterior from C57BL/6J male and female mice, fed on a high-fat, high-sugar diet (HFHS) for 4 and 20 weeks starting at 3 weeks of age. Results: Experimental data revealed different HFHS-associated transcriptional patterns in female and male mice, within a background of sex-associated transcriptional differences across muscle maturation. Conclusions: These preliminary observations lay the groundwork for further investigation of the mechanisms underlying the differences in diet-associated transcriptional patterns between female and male skeletal muscle, with the ultimate goal of addressing obesity and its comorbidities.

## 1. Introduction

Childhood obesity and overweight represent a paramount problem worldwide. As reported by UNICEF, in 2025, the levels of obesity in school-aged children and adolescents surpassed that of underweight (https://www.unicef.org/reports/feeding-profit, accessed on 3 July 2026). The key factors that have determined the rise in obesity can be found in the uncontrolled ingestion of energy-dense foods rich in fats and sugars and sugar-sweetened beverages and the lack of adequate physical activity, which together constitute an “obesogenic environment” [1].

Pediatric obesity represents a major concern for global health because it can affect not only the actual quality of life of young individuals but can also have a dramatic impact on their future health. In fact, because children are still undergoing growth and developmental processes, their physiological systems are particularly susceptible to environmental and metabolic disturbances. Beyond obvious metabolic impairment, pediatric obesity carries complications that, long term, can result in cardiovascular diseases, metabolic dysfunction-associated steatotic liver disease, polycystic ovarian syndrome, kidney and thyroid dysfunction, asthma, immunologic and dermatologic disorders, psychological problems, significant increases in adiposity, and skeletal muscle dysfunction [2,3,4,5].

Indeed, skeletal muscle assumes a prominent role in both the development and management of obesity because, being 40% of body mass, it is responsible for 80% of insulin-stimulated glucose uptake [6]. In this context, it has been suggested that obesity can lead to a domino effect that can contribute to the establishment of a multidimensional condition that shares several similarities with aging [5,7].

Both adipose and skeletal muscle tissue structure and physiology depend on biological sex. From the fat metabolism side, the prevalence of obesity among men is higher than in women [8]. Nevertheless, women have more body fat than men, which is mainly stored at the gluteal–femoral region, while men store more fat in the intra-abdominal region [9]. Interestingly, in the peri- and post-menopausal period, fat redistributes to the intra-abdominal region in women similarly to men, increasing the probability of developing obesity and associated morbidities [10,11,12]. Also, skeletal muscle form and function present sex-dependent differences. In fact, recent research reports growing evidence on the presence of sex-specific differences during development, homeostasis, adaptive responses, and diseases [13,14,15]. In both sexes, skeletal muscles have 3000 differentially expressed genes [16], different hormone regulation [17], and distinct fiber-type composition [16], which contribute to different physiological responses in normal conditions and upon age-dependent wasting [14].

In addition to sex-dependent differences, it is important to remember that skeletal muscle is a highly plastic tissue, and form and function are controlled by precise genetic networks and signaling pathways that can change during the life course, dictating different physiological responses [18]. As a result, the skeletal muscle landscape is rather complex, as gene expression and the response to physiological and non-physiological events depend on sex, age, diet, exercise, and many other factors. Despite the increasing interest in sex-dependent development and the impact of obesity on adult skeletal muscle, both in human and animal models, scarce information in pediatric and early adulthood stages has been reported [4]. Although previous studies have investigated the effects of high-fat diets on skeletal muscle, including studies addressing sex differences or specific developmental stages, the combined analysis of early high-fat dietary exposure in juvenile mice of both sexes and the resulting skeletal muscle transcriptomic response remains mainly uncharacterized.

Aiming at understanding the early alterations in skeletal muscle tissue induced by energy-dense foods rich in fats and sugars and sugar-sweetened beverages during maturation in mice, we recently explored a mouse model of juvenile obesity induced by a high-fat, high-sugar (HFHS) diet. This diet-induced obese model is characterized by an increase in visceral adipose weight, increased liver weight, dyslipidemia, insulinemia, Homeostasis Model Assessment Insulin Resistance (HOMA-IR), and hepatic steatosis, with inflammation and fibrosis consistent with the development of metabolic dysfunction-associated fatty liver disease (MAFLD) [19,20]. Specifically, we noticed significant differences in the HFHS diet vs. age-matched controls in total cholesterol, High-Density Lipoprotein, and Low-Density Lipoprotein (HDL, LDL), accompanied by initial hepatic steatosis and hepatic inflammation at 4 weeks of the diet, progressing at 20 weeks of the diet to significant differences in total cholesterol, HDL, LDL, Alanine Amino Transferase (ALT), Aspartate Amino Transferase (AST), hepatic steatosis, inflammation, and ballooning [21]. Nevertheless, 20 weeks of HFSH conditions represent a clinically reversible stage. Interestingly, at 20 weeks of the diet, we reported sex-dependent modifications in both the Body Mass Index (BMI) and HOMA-IR in males compared with females and sex-dependent alterations in skeletal muscles of obese mice: an increase in type II fibers, mitochondrial activity, and lipid content was observed in males, while capillary density was altered in females [22].

The above-mentioned phenotypic differences observed between female and male mice exposed to the HFHS diet in juvenile mice [19,20,21,22], together with the limited understanding of the molecular mechanisms underlying these differences, prompted us to investigate the transcriptional response of skeletal muscle to HFHS exposure in male and female mice. With this aim, we analyzed the transcriptome of the tibialis anterior muscle from male and female mice at 4 weeks of the HFHS diet, to capture the initial changes associated with disease development, and at 20 weeks of the HFHS diet, which represents an early defined but still clinically reversible stage.

From the transcriptional perspective, our data suggest the presence of an early sex-specific dimorphism in the skeletal muscle tissue accompanied by a dominant maturation axis, where HFHS exposure interferes with selective processes that only partially converge with maturation-associated remodeling, leading us to propose that diet responsiveness depends on the biological context in which it occurs. These observations represent a further step toward understanding the mechanisms of the dimorphic response to diet during maturation and could provide key concepts for understanding the impact of obesogenic diets by sex and timeframe, possibly laying the groundwork to modulate our approach to obesity and comorbidities in the early stages of life.

## 2. Materials and Methods

### 2.1. Animals and Diet

C57BL/6J (JAX^®^) breeding mice were originally obtained from Charles River Laboratories (Calco, Italy). Pups used for experimental procedures were bred and housed under specific pathogen-free (SPF) conditions at the animal facility of the University of Trieste (Trieste, Italy). At 3 weeks of age, animals, individually identified by ear punching, were randomly group-housed in cages in a temperature-controlled environment (22 ± 2 °C) on a 12 h light/dark schedule and fed ad libitum with a control diet (CTRL, Special Diets Services (SDS) 811,900 VRF1, Witham, UK) or a high-fat, high-carbohydrates diet (HFHS, Surwit diet, D12331, Research Diets, New Brunswick, NJ, USA), combined with 42 g/L of fructose/sucrose in drinking water, either for 4 or for 20 weeks [21,22]. The standard chow diet contained 19.1 gm% crude protein, 4.8 gm% crude fat, 3.9 gm% crude fiber, and 6.2 gm% crude ash (10% mixture), with an energy distribution of 22.3 kcal% from protein, 65.3 kcal% from carbohydrates, and 12.4 kcal% from fat, for a total calculated energy density of ~3.38 kcal/g. The purified Surwit diet contained 23 gm% protein, 35.8 gm% fat, and 35.5 gm% carbohydrates, providing 16.4 kcal% from protein, 58.0 Kcal% from fat, and 25.5 kcal% from carbohydrates, with an energy density of 5.56 kcal/g. The diets were selected to represent two distinct nutritional conditions: the CTRL diet, routinely used in the SPF animal facility, was chosen to provide a nutritionally balanced regimen supporting the health and well-being of the housed animals, whereas the HFHS diet was designed to mimic a “junk diet” characterized by a high intake of calories from saturated fats and sugars, including sugar-sweetened beverages. At the end of the treatment, animals were anesthetized with an anesthetic overdose of ketamine/xylazine (40–10 mg/kg) and sacrificed by decapitation. At the time of sacrifice, animals were 7 and 23 weeks old, depending on the length of the diet protocol. No animals died or reached the predefined humane endpoint (HEP) criteria requiring euthanasia during the experimental period. The only overt phenotypic alterations observed were those described in the Introduction Section and expected as part of the obesity phenotype induced by the experimental model. The experimental protocol was approved by the animal care and use committee of the University of Trieste (OPBA: Organismo Per il Benessere Animale) and the competent Italian Ministry (# 56/2022PR). All the procedures were performed according to the Italian Law (D.Lgs.26/2014) and the European Community Directive (2010/63/EU). The distress score evaluation was performed throughout the experimental period. For transcriptomic analyses, each of the eight (2 sex × 2 diet × 2 age) groups included four independent mice (32 animals total; 16 females and 16 males). The balanced design across sex, diet, and age was selected while limiting animal use in accordance with the 3R principle.

### 2.2. Body Mass Index (BMI), Glucose, and Triglyceride Content in Blood

The 3-week-old female and male mice were exposed to 4 and 20 weeks of the control or HFHS diet. At the time of sacrifice, animals were 7 and 23 weeks old, depending on the length of the diet protocol. Body weight and naso-anal length were measured after sacrifice to calculate BMI. Significant differences among groups were determined by performing two-way ANOVA followed by Šídák’s multiple-comparisons test, with GraphPad Prism version 11.02, GraphPad Software (La Jolla, CA, USA). Intracardiac sampling of the blood (after 6 h fasting) was performed to measure glycemia. Glucose was measured in whole blood using One Touch verio IQ1 m (Life Scan Europe, Zug, Switzerland) according to the manufacturer’s instructions [19]. An enzymatic colorimetric kit (Roche Diagnostics GmbH; Mannheim, Germany) was used to measure triglycerides in a Roche HITACHI Cobas e501 instrument (Roche Diagnostics GmbH; Mannheim, Germany) according to the manufacturer’s instructions [19]. Significant differences among groups in BMI, fasting glucose, and triglyceride levels were determined by performing Student’s *t*-test with GraphPad Prism version 11.02, GraphPad Software (La Jolla, CA, USA).

### 2.3. Tissue Processing

Tibialis anterior, a mixed-fiber-type skeletal muscle that presents fibers mainly expressing Myosin Heavy Chain IIA, IIX, and IIB [23], was selected as a representative, metabolically responsive hindlimb muscle that combines a predominantly fast-fiber phenotype with an oxidative component, and that, meanwhile, provides an adequate amount of tissue for high-quality RNA extraction. Tibialis anterior muscles were dissected, rinsed in PBS, allocated into vials, snap-frozen in liquid nitrogen, and preserved at −80 °C. Left and right tibialis anterior muscles from each mouse were pooled (approximately 80–100 mg total) for RNA extraction and shipped to Novogene on dry ice.

### 2.4. Bulk mRNA Sequencing of RNA Muscle Samples

Total RNA was extracted from muscle tissue and quality assessed using the Agilent 5400 fragment analyzer (Agilent Technologies, CA, USA). Only RNA samples with integrity number (RIN) values > 7.0 were used for library preparation. Sequencing libraries were prepared by Novogene following a poly-A-selected mRNA workflow. Briefly, mRNA was enriched from total RNA using poly-T oligo-attached magnetic beads, ethanol-precipitated, fragmented by sonication, and reverse-transcribed into first-strand cDNA using random hexamer primers. Second-strand cDNA synthesis was then performed, followed by end repair, A-tailing, adapter ligation, size selection (250–300 bp), PCR amplification, and purification using the AMPure XP system (Beckman Coulter, Beverly, MA, USA). Library concentration and quality were assessed using Qubit, qPCR, and size distribution detected with a fragment analyzer. Qualified libraries were pooled according to their effective concentration and expected data output and sequenced on the Illumina NovaSeq X Plus platform (Illumina, San Diego, CA, USA) using 150 bp paired-end reads, with a sequencing depth of ≥40 million read pairs per sample. Raw reads were filtered to remove adapters and low-quality bases. Clean paired-end reads were aligned to the Mus Musculus (GRCm39/mm39) reference genome using HISAT2 v2.0.5. Gene-level read counts were obtained using featureCounts v1.5.0-p3 based on the corresponding gene annotation file. Gene expression levels were estimated as fragments per kilobase of transcript per million mapped reads (FPKM), accounting for sequencing depth and gene length. Raw gene-level counts were used for downstream differential expression analysis. Differential expression analysis was performed using the DESeq2 1.20.0 R package, which models count data based on the negative binomial distribution.

### 2.5. Statistical Analysis of Differential Gene Expression and Functional Enrichment

To identify differentially expressed genes (DEGs) associated with sex, HFHS exposure, and maturation, three sets of pairwise comparisons were performed. First, sex-associated transcriptional differences under control conditions were assessed by comparing female and male CTRL mice at 7 and 23 weeks of age. Second, HFHS-associated responses were assessed separately by sex after 4 weeks of exposure (7 weeks of age) and 20 weeks of exposure (23 weeks of age), each against age- and sex-matched CTRL mice. Third, maturation-associated changes were assessed within each sex by comparing 23-week-old with 7-week-old CTRL mice. For all comparisons, *p*-values were adjusted using the Benjamini–Hochberg procedure to control the false discovery rate (FDR). Genes were defined as significantly differentially expressed at adjusted *p* < 0.05 and |log2 fold change| > 0.5. The |log2 fold change| > 0.5 threshold was selected to retain moderate transcriptional changes while still requiring FDR-adjusted statistical significance.

Functional enrichment analysis of DEGs was performed using g:Profiler (https://biit.cs.ut.ee/gprofiler/; accessed 4 November 2025). Ensembl gene IDs of DEGs were used as input in the g:GOST functional profiling interface, with Mus musculus selected as the organism. The g:SCS method was used as the significance threshold, with a user-defined *p*-value threshold of 0.05. Gene Ontology terms, together with KEGG and Reactome pathways, were included in the analysis. When applicable, enrichment analyses were conducted separately for up-regulated and down-regulated genes to preserve direction-specific biological interpretation. Functional enrichment was also applied to unique and overlapping DEG subsets derived from the intersection analyses of maturation- and diet-associated comparisons. Venn diagrams were obtained using the InteractiveVenn web-based tool [24]. Violin plots showing expression counts of the most interesting, intersected genes were generated using GraphPad Prism 11.02. The median and interquartile range are indicated in each plot.

## 3. Results

### 3.1. Body Weight, Body Mass Index (BMI), Blood Glucose, and Triglyceride Levels After 4 and 20 Weeks of the HFHS Diet

**Figure 1:**
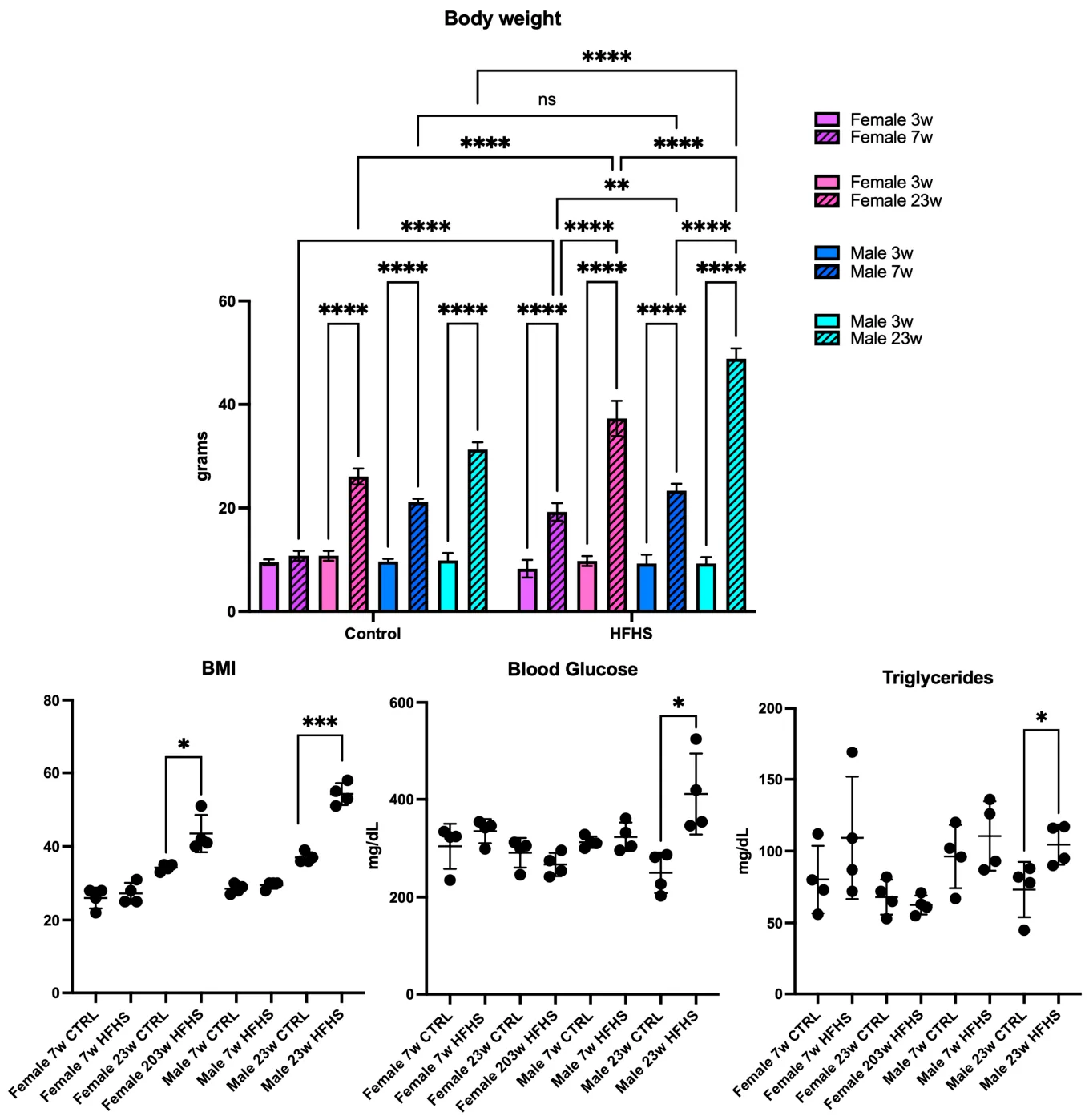
Body weight, BMI, fasting glucose, and triglycerides of standard chow (control) and HFHS-fed mice. Data are reported as mean ± SD (significantly different compared to age-matched and sex-matched controls; * *p* < 0.05, ** *p* < 0.01, *** *p* < 0.0005, **** *p* < 0.0001; n = 4).

The measurement of body weight showed that after 4 (corresponding to the age of 7 weeks) and 20 weeks (corresponding to the age of 23 weeks) of the HFHS diet, there was an increase in both females and males (Figure 1, top panel). However, the effect on BMI was significantly impacted only after 20 weeks of HFHS feeding, when the BMI increase was about 25% in females and 45% in males. Blood glucose and triglycerides displayed a significant increase only in males after 20 weeks of HFHS feeding (corresponding to 23 weeks of age, Figure 1, bottom panels).

### 3.2. Sex-Specific Transcriptomic Landscape of Tibialis Anterior Muscle at 7 and 23 Weeks of Age

**Figure 2:**
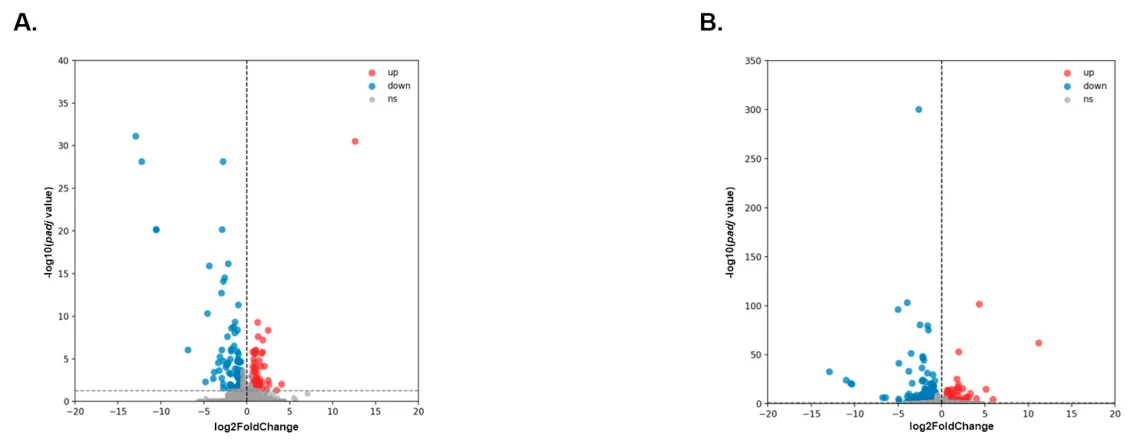
Sex-specific transcriptomic differences in the tibialis anterior muscle across developmental stages. Volcano plots showing differentially expressed genes (DEGs) between female vs. male mice at (**A**) 7 weeks (peri-pubertal) and (**B**) 23 weeks (sexually mature). Each point represents a gene plotted by log_2_ fold change (*x*-axis) and –log_10_(adjusted *p*-value) (*y*-axis). Up-regulated genes are shown in red, down-regulated genes in blue, and non-significant genes in gray. The horizontal dashed line indicates the significance threshold (FDR = 0.05), and the vertical dashed line marks log_2_FC = 0. Positive log_2_FC values indicate higher expression in females relative to males, whereas negative log_2_FC values indicate lower expression in females relative to males.

To define sex-dependent transcriptional differences under control conditions, we compared female and male CTRL mice at 7 weeks (peri-pubertal) and at 23 weeks (sexually mature) (Figure 2 and Supplementary Materials Table S1).

**Figure 3:**
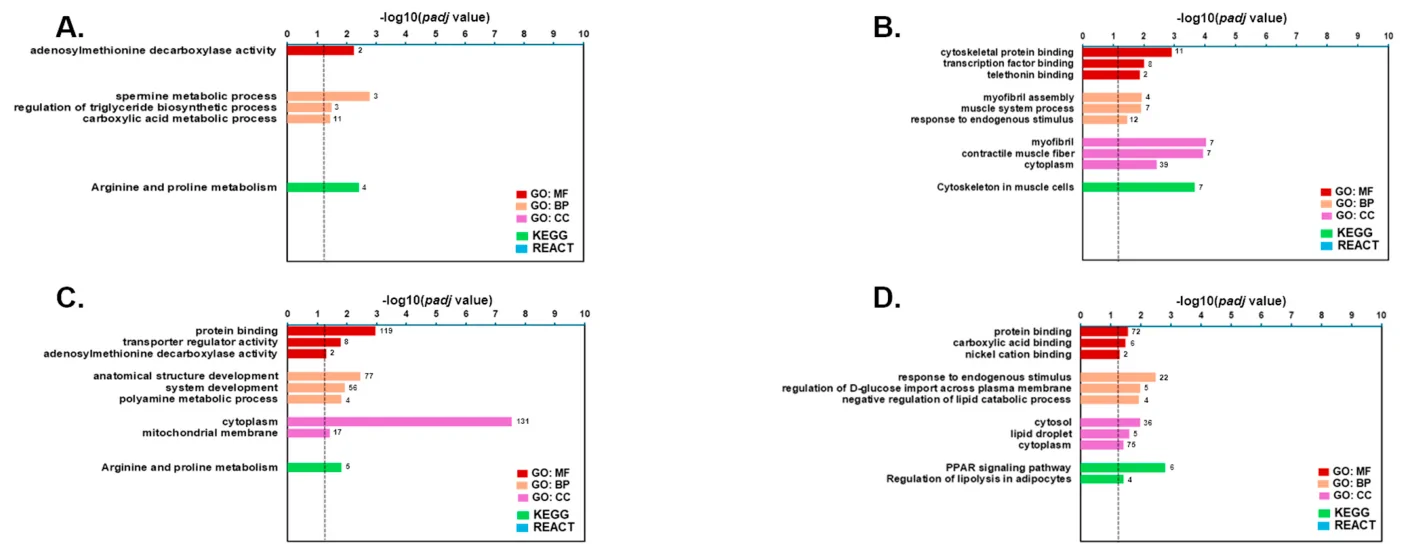
Over-representation analysis of DEGs in muscle transcriptomes of female vs. male mice. Horizontal bar plots show enriched Gene Ontology (GO) terms and pathways identified using g:Profiler at 7 weeks (**A**,**B**) and 23 weeks (**C**,**D**). The top three enriched GO categories (GO:MF, GO:BP, GO:CC) and KEGG and Reactome pathways are shown for down-regulated (**A**,**C**) and up-regulated genes (**B**,**D**). The *x*-axis represents −log_10_(adjusted *p*-value); the dashed line indicates significance (*p*Adj ≤ 0.05, g:SCS correction). Numbers next to bars indicate the intersection size (number of overlapping genes). GO:MF, Gene Ontology Molecular Function; GO:BP, Gene Ontology Biological Process; GO:CC, Gene Ontology Cellular Component; KEGG, Kyoto Encyclopedia of Genes and Genomes; REACT, Reactome Pathway Database.

At 7 weeks, 168 genes were differentially expressed between the sexes (98 genes down-regulated and 70 genes up-regulated), evidencing sexual dimorphism at the peri-pubertal stage (Figure 2A). The functional ontological enrichment of down-regulated expressed genes from the female vs. male comparison indicated a compact signature centered on spermine/polyamine-associated metabolic terms, consistent with the expected sex-related contribution of these pathways. Conversely, up-regulated terms were dominated by muscle-structural and cytoskeletal categories, including myofibril assembly, muscle system process, sarcomere, A band, I band, and cytoskeleton in muscle cells, among other ontological terms (Figure 3A,B, Table S2).

At 23 weeks, 406 DEGs were identified (249 lower and 157 higher in females; Figure 2B), indicating more extensive sex-associated transcriptional differences than at 7 weeks. Genes lower in females remained enriched for spermine/polyamine metabolism and included arginine and proline metabolism together with developmental and differentiation-associated processes (Figure 3C; Table S3).

Conversely, genes up-regulated in females showed a distinct metabolic and signaling profile. Enriched terms included glucose and carbohydrate imports across the plasma membrane, regulation of D-glucose transport, lipid catabolic processes, lipid droplet-related terms, and regulation of lipolysis. The enrichment of PPAR signaling and regulation of lipolysis in adipocytes further supports a female-biased metabolic program involving lipid handling and substrate utilization (Figure 3D, Table S3). Overall, the 23-week sexually dimorphic signature suggests a divergence between a male-associated polyamine/remodeling profile and a female-associated metabolic regulatory profile (full g:Profiler enrichment results in Tables S2 and S3).

Together, these findings demonstrate that sexual dimorphism in skeletal muscle is both established early and substantially reinforced with maturation, providing critical physiological levels that likely shape subsequent responses to dietary intervention.

### 3.3. Sex-Stratified Transcriptional Responses of Skeletal Muscle to the HFHS Diet

**Figure 4:**
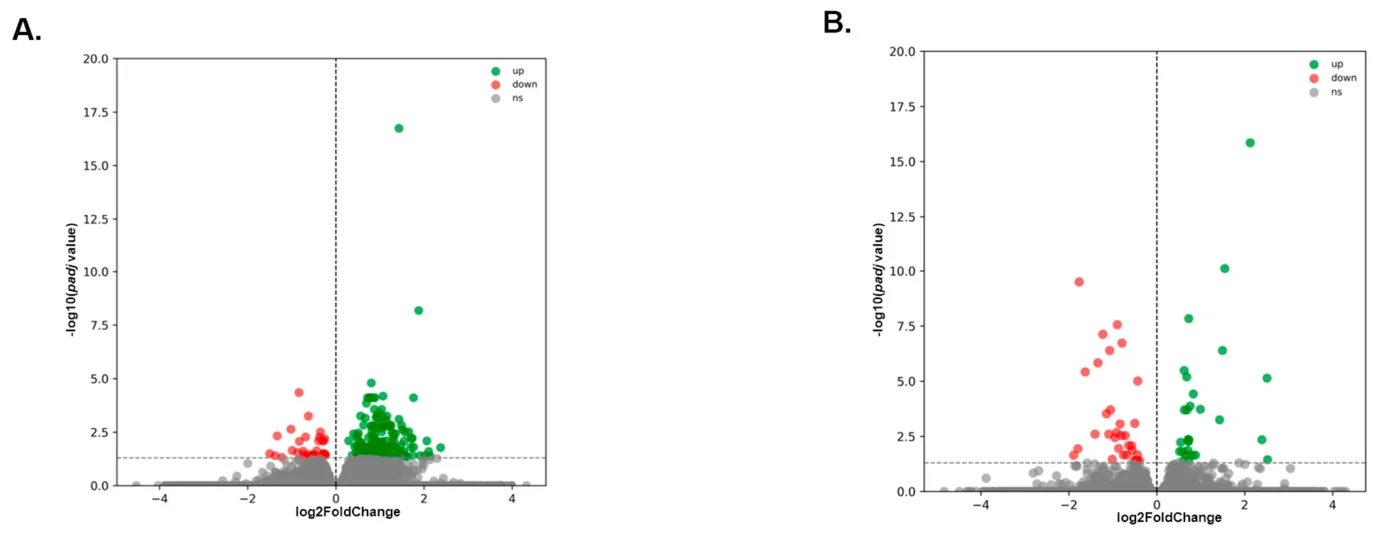
Transcriptome profile of the tibialis anterior muscle from female mice fed the HFHS diet. Volcano plot showing DEGs after (**A**) 7 weeks and (**B**) 23 weeks on the HFHS diet. Each point represents a gene plotted by log_2_ fold change (*x*-axis) and −log_10_(adjusted *p*-value) (*y*-axis). Up-regulated genes are shown in green, down-regulated genes in red, and non-significant genes in gray.

Sex-stratified analysis revealed different transcriptional patterns following HFHS exposure at the two examined time points. In females, the response was more pronounced at the early time point, as illustrated by volcano plots (Figure 4; Table S4), which showed a broader distribution of significant transcripts at 7 weeks in the HFHS group (184 DEGs; 164 up-regulated and 20 down-regulated; Figure 4A) compared with 23 weeks. At the later time point, the transcriptional profile was markedly more restricted, with only 55 DEGs identified (27 up-regulated and 28 down-regulated; Figure 4B), indicating reduced transcriptional responsiveness over time.

**Figure 5:**
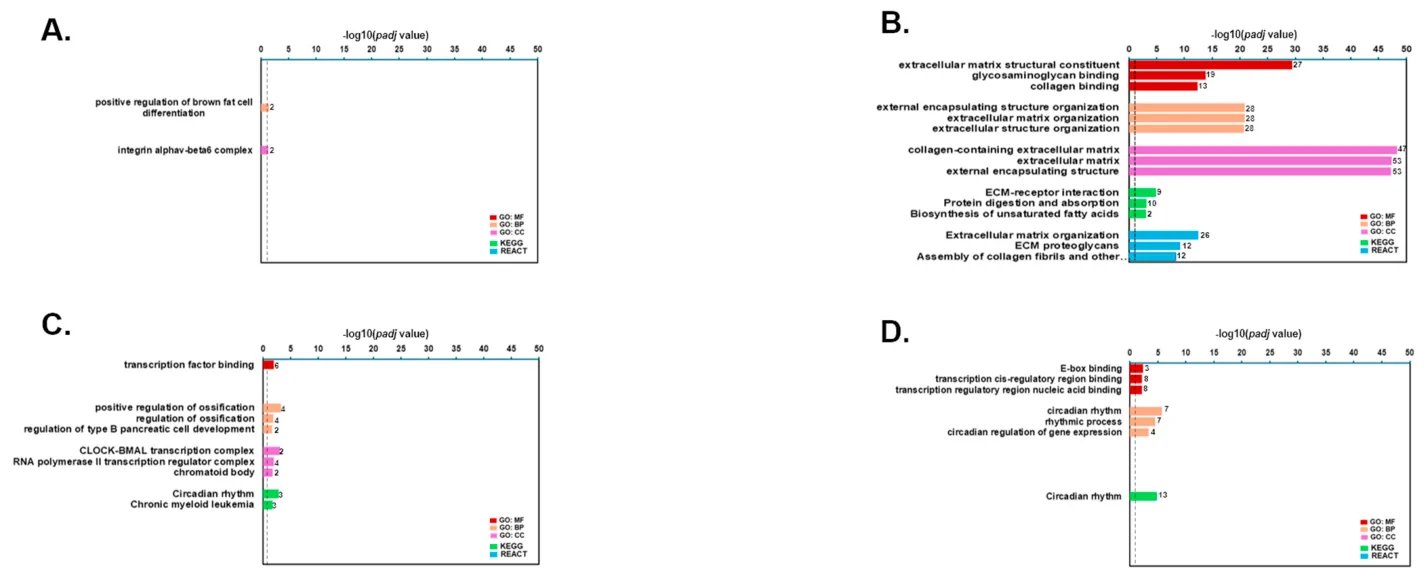
Over-representation analysis of DEGs in muscle transcriptomes from female mice on the HFHS diet. Horizontal bar plots show GO terms and pathways identified using g:Profiler after 7 weeks (**A**,**B**) and 23 weeks (**C**,**D**) of HFHS feeding.

At week 7, functional enrichment analysis revealed that early diet-responsive genes in females were primarily associated with extracellular matrix (ECM) organization and structural remodeling, including gene ontologies like extracellular matrix structural constituents, glycosaminoglycan binding, collagen-containing extracellular matrix, ECM proteoglycans, and collagen fibril assembly (Figure 5B, Table S5). Down-regulated genes at this stage were linked to brown fat cell differentiation, adipogenesis, and the integrin αVβ3 complex (Figure 5A, Table S5).

By 23 weeks on the HFHS diet, the transcriptional response in females shifted both in magnitude and functional profile. The limited number of DEGs identified at this stage was primarily associated with circadian and transcriptional regulatory pathways. Down-regulated genes were enriched for circadian rhythm, CLOCK: BMAL1 transcription complex, chromatin organization, and transcription factor binding. At the same time, up-regulated genes were also linked to circadian regulation of gene expression and rhythmic processes (Figure 5C,D, Table S6).

**Figure 6:**
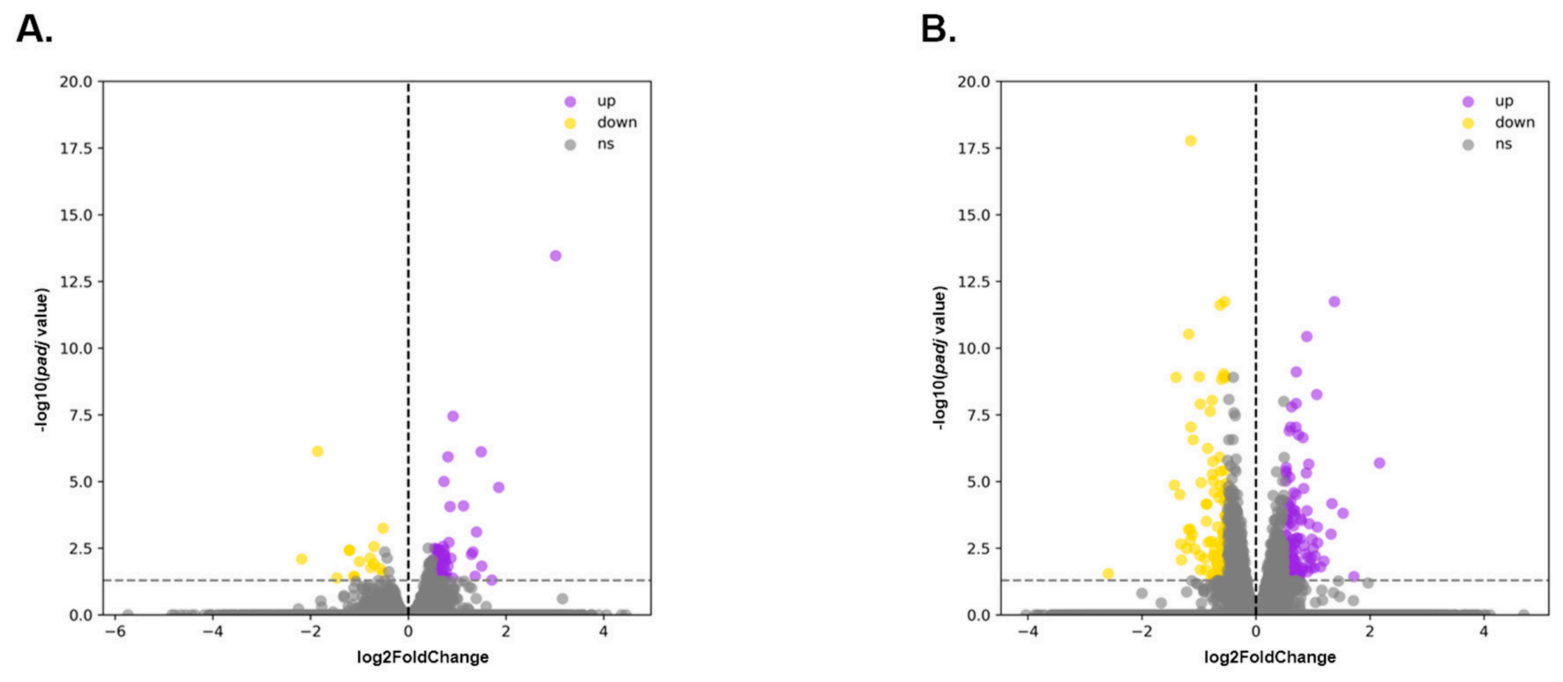
Transcriptome profile of tibialis anterior muscle from male mice fed the HFHS diet. Volcano plot showing DEGs after (**A**) 7 weeks and (**B**) 23 weeks on the HFHS diet. Each point represents a gene plotted by log_2_ fold change (*x*-axis) and −log_10_(adjusted *p*-value) (*y*-axis). Up-regulated genes are shown in violet, down-regulated genes in yellow, and non-significant genes in gray.

In contrast, male mice exhibited a more progressive transcriptional response to dietary challenge. Volcano plots (Figure 6, and Table S7) revealed relatively few DEGs after 4 weeks of HFHS feeding (7 weeks of age; 79 DEGs; 64 up-regulated and 15 down-regulated; Figure 6A), whereas a much stronger response was observed after 20 weeks of HFHS feeding (23 weeks of age), with a substantial increase in both up-regulated and down-regulated genes (231 DEGs; 125 up-regulated and 106 down-regulated; Figure 6B).

**Figure 7:**
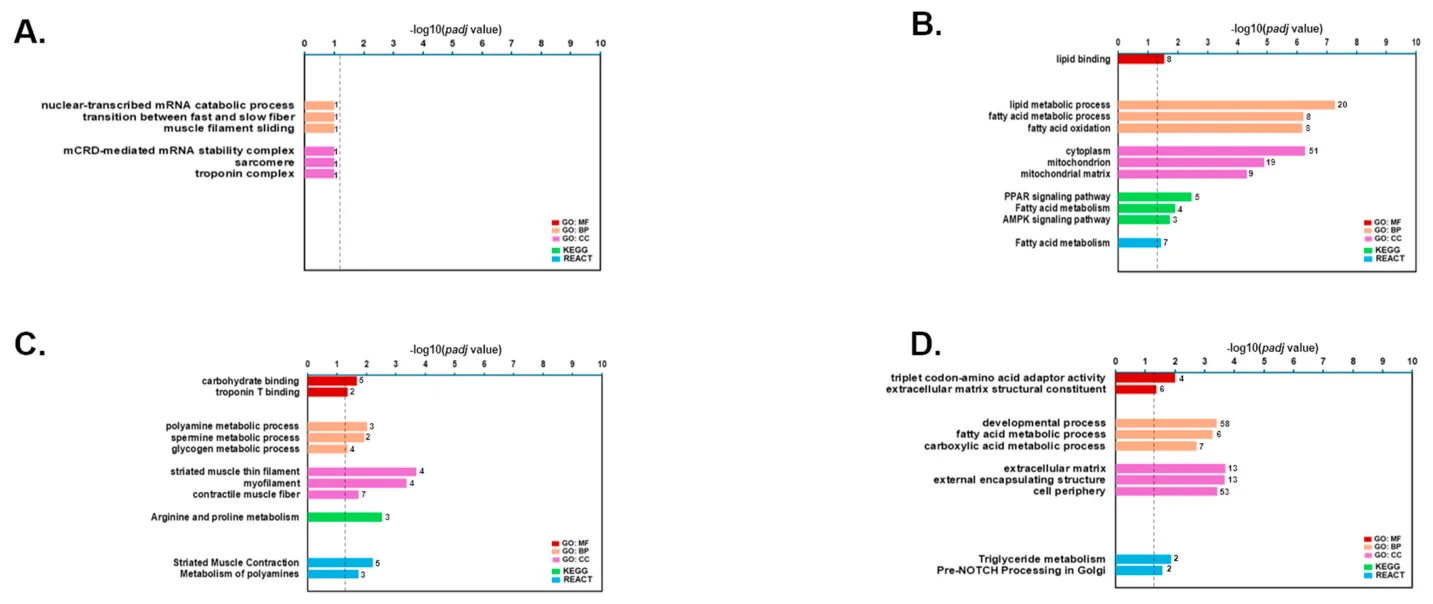
Over-representation analysis of DEGs in muscle transcriptomes from male mice on the HFHS diet. Horizontal bar plots show GO terms and pathways identified using g:Profiler at 7 weeks (**A**,**B**) and 23 weeks (**C**,**D**) of HFHS feeding. The top three enriched GO categories (GO:MF, GO:BP, GO:CC) and KEGG and Reactome pathways are shown for down-regulated (**A**,**C**) and up-regulated genes (**B**,**D**).

At 7 weeks in the HFHS group, down-regulated genes in males were associated with processes such as muscle filament sliding, fiber-type transition, and mRNA catabolic processes, although these pathways did not reach significance in enrichment analysis (Figure 7A, Table S8). In contrast, up-regulated genes were enriched for fatty acid metabolism, fatty acid oxidation, and PPAR signaling pathways, indicating early activation of lipid handling and oxidative metabolism (Figure 7B, Table S8).

By 23 weeks, the transcriptional response in males expanded considerably and encompassed multiple biological processes. Down-regulated genes were enriched mainly in cellular component ontologies, including striated muscle contraction, muscle filament sliding, muscle structure development, and biological process ontologies related to polyamine and glycogen metabolism. Conversely, up-regulated genes were associated with developmental processes, fatty acid metabolism, extracellular matrix organization, collagen-related pathways, triglyceride metabolism, and pre-NOTCH processing (Figure 7C,D, Table S9).

### 3.4. Maturation-Dependent Transcriptional Remodeling Dominates over Dietary Effects

**Figure 8:**
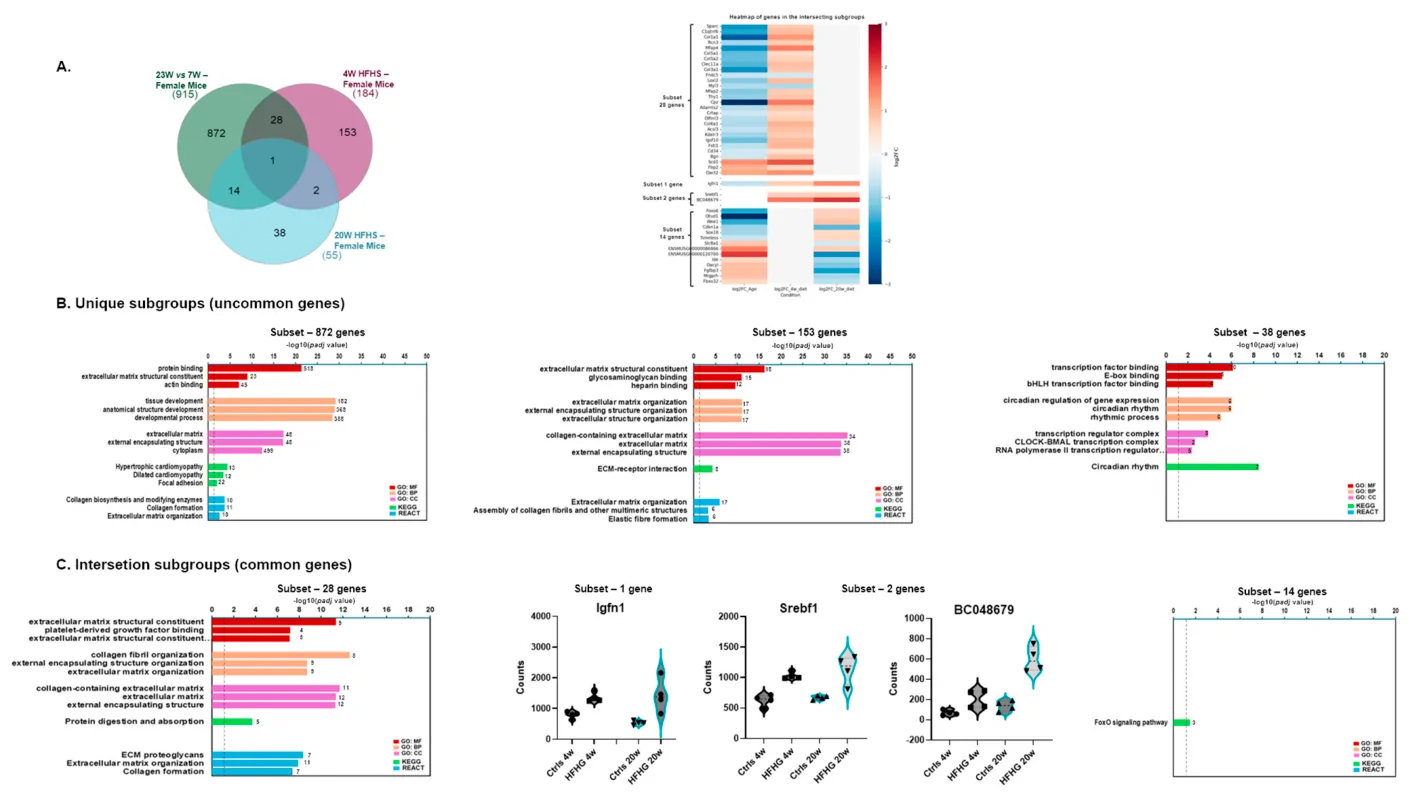
DEGs in skeletal muscle of female mice across maturation and HFHS diet conditions. (**A**) Venn diagram showing DEGs from three comparisons: maturation effect (23 W vs. 7 W), early diet effect (7 W HFHS vs. 7 W ctrls), and late diet effect (23 W HFHS vs. 23 W ctrls). Heatmap of shared genes across conditions. Heatmap showing log2 fold-change values for genes appearing in the intersection of the three comparisons (28-gene subset, 1 shared gene, 2 shared genes, and 14-gene subset). Group separators highlight distinct expression patterns linked to age and dietary perturbations. (**B**) Unique subgroups (uncommon genes). Gene ontology and functional enrichment results for unique gene subsets of the Venn diagram (872, 153, and 38 genes) are displayed by the horizontal bar charts (GO: MF, GO: BP, GO: CC, KEGG, and Reactome). Bar plots represent the most significantly enriched biological terms ranked by −log10 (adjusted *p*-value). (**C**) Intersection subgroups (common genes). Gene ontology and functional enrichment results for the intersection gene sets (28 shared genes and a 14-gene subset). Violin plots illustrate normalized expression counts for the intersecting sets of genes (Igfn1, Srebf1, BC048679) across all experimental groups, which did not show any functional enrichment.

**Figure 9:**
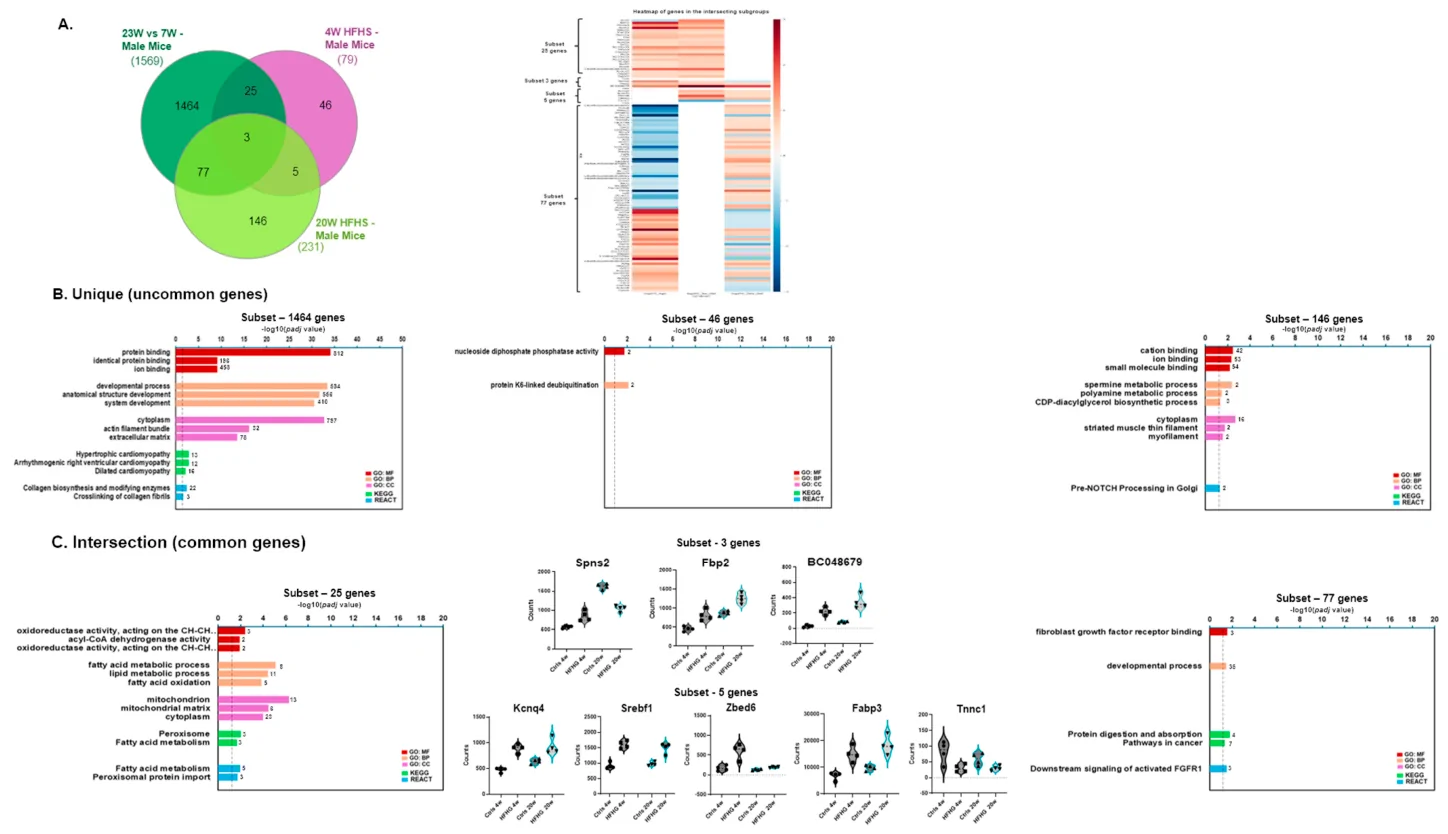
DEGs in skeletal muscle of male mice across maturation and HFHS diet conditions. (**A**) Venn diagram showing DEGs from three comparisons: maturation effect (23 W vs. 7 W), early diet effect (7 W HFHS vs. 7 W ctrls), and late diet effect (23 W HFHS vs. 23 W ctrls). Heatmap of shared genes across conditions. Heatmap showing log2 fold-change values for genes appearing in the intersection of the three comparisons (25-gene subset, 3 shared genes, 5 shared genes, and 77-gene subset). Group separators highlight distinct expression patterns linked to age and dietary perturbations. (**B**) Unique subgroups (uncommon genes). Gene ontology and functional enrichment results for unique gene subsets of the Venn diagram (1464, 46, and 146 genes) are displayed by the horizontal bar charts (GO: MF, GO: BP, GO: CC, KEGG, and Reactome). Bar plots represent the most significantly enriched biological terms ranked by −log10(adjusted *p*-value). (**C**) Intersection subgroups (common genes). Gene ontology and functional enrichment results for the intersection gene sets (25 shared genes and a 77-gene subset). Violin plots illustrate normalized expression counts for the intersecting sets of genes (Spns2, Fbp2, BC048679, Kcnq4, Srebf1, Zbed6, Tnnc1) across all experimental groups, which did not show any functional enrichment.

To determine which transcriptional changes were shared or comparison-specific, we intersected DEG sets from maturation (23- versus 7-week CTRL), the early HFHS response (4 weeks of exposure; 7-week HFHS versus CTRL), and the prolonged HFHS response (20 weeks of exposure; 23-week HFHS versus CTRL) separately in each sex (Figure 8 and Figure 9).

In females (Figure 8A), the Venn diagram shows that most DEGs were comparison-specific, with large unique gene sets associated with maturation (872 genes), early diet response (153 genes), and late diet response (38 genes), with only limited overlap between conditions. Enrichment of these unique subsets broadly recapitulated the comparison-specific results, with ECM, cytoskeletal, and muscle-structural terms prominent in maturation and early HFHS subsets and transcriptional/circadian terms in the prolonged HFHS subset (Figure 8B).

Despite these distinct comparison-specific signatures, only limited overlap was detected among female DEG sets (Figure 8A, Venn diagram). In total, 28 genes were shared between maturation and the early HFHS response, 14 genes were shared between maturation and the late HFHS response, two genes were shared between early and late HFHS responses, and only one gene, Igfn1, was common to all three comparisons. The 28-gene intersection showed enrichment for ECM-related terms, whereas the 14-gene intersection was associated only with FoxO signaling; the one- and two-gene intersections (*Igfn1*; *Srebf1*, *BC048679*) were not suitable for robust pathway-level interpretation (Figure 8C).

A similar but more pronounced pattern was observed in males (Figure 9A, Venn diagram). Maturation again accounted for the large DEG set (1464 genes), followed by a substantial late diet response (146 genes) set and a relatively modest early response (46 genes) set. The maturation-specific subset was enriched for structural, cytoskeletal, and developmental terms, whereas the prolonged HFHS-specific subset included metabolic, contractile, and signaling-related processes (Figure 9B).

The male intersecting subsets were also relatively small compared with the unique age-associated gene set (Figure 9A, Venn diagram, central panel). In total, 25 genes were shared between maturation and early HFHS response, 77 genes were shared between maturation and late HFHS response, 5 genes were shared between early and late HFHS responses, and 3 genes were common to all comparison groups. The 25-gene intersection was enriched for oxidative and fatty acid metabolic processes. The larger 77-gene intersection between maturation and late HFHS response showed enrichment for fibroblast growth factor receptor binding, developmental processes, extracellular structure organization, and downstream signaling of activated FGFRs (Figure 9C). The three- and five-gene intersections, represented by genes including Spns2, Fbp2, BC048679, Kcnq4, Srebf1, Zbed6, Fabp3, and Tnnc1, did not support broad pathway-level conclusions and instead highlight small gene-specific points of overlap.

### 3.5. Integrated Overview of Sex-, Maturation-, and HFHS-Associated Transcriptional Patterns

**Figure 10:**
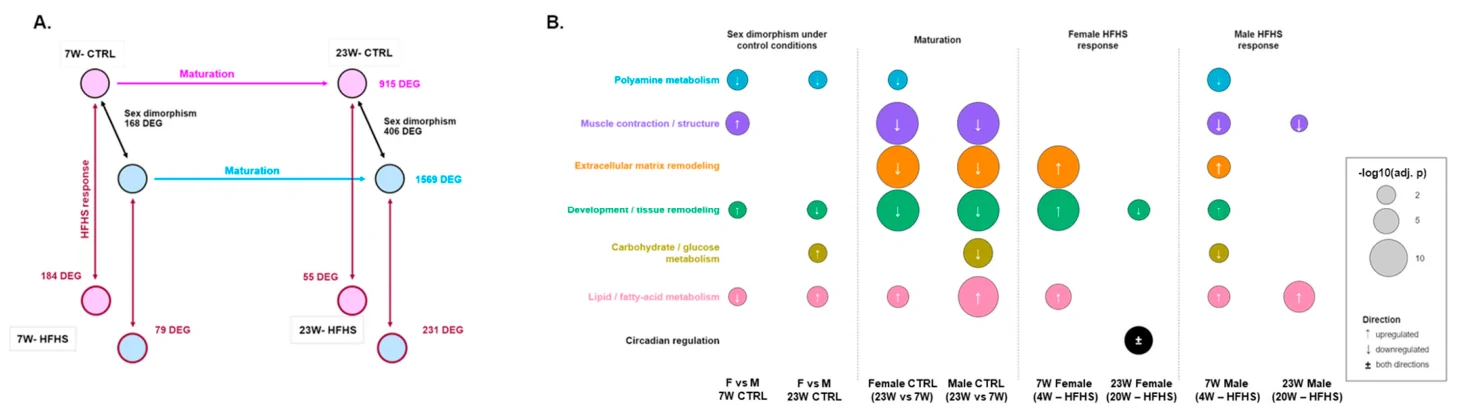
Integrated overview of the major transcriptomic patterns associated with sex, maturation, and HFHS exposure in skeletal muscle. (**A**) Summary of the transcriptomic comparisons performed in female and male mice under control and HFHS conditions. Numbers indicate the differentially expressed genes (DEGs) identified in the sex-associated, maturation-associated, and HFHS-associated comparisons. Pink and blue indicate female and male mice, respectively. (**B**) Bubble matrix summarizing selected recurrent biological themes identified by functional enrichment analysis across transcriptomic comparisons. Columns indicate sex-associated differences under control conditions, maturation-associated changes between 23- and 7-week-old control mice, and HFHS-versus-control responses after 4 and 20 weeks of dietary exposure. Rows indicate curated biological themes derived from significant g:Profiler terms. Bubble size is proportional to −log10 (adjusted *p*-value), and colors identify biological themes. Arrows indicate enrichment among up-regulated (↑) or down-regulated (↓) DEGs; ± indicates related enriched terms detected in both directional DEG sets.

To provide an integrated overview of the transcriptional patterns identified across the different analyses, the magnitude and functional characteristics of the major transcriptomic responses are summarized in Figure 10 (Table S10). The comparison scheme highlights the distinct contributions of maturation and HFHS exposure in female and male skeletal muscle, whereas the bubble matrix summarizes the main biological programs associated with sex, maturation, and dietary response. Together, these analyses show that maturation represents a major transcriptional component in both sexes, while the sex-stratified HFHS comparisons reveal different transcriptional patterns across the two exposure durations, including an early matrix/remodeling signature in females and a broader late metabolic and structural response in males.

## 4. Discussion

Prompted by our previous phenotypic observations in this juvenile HFHS model [19,20,21,22], we investigated the effect of an HFHS diet on the transcriptomic profile of tibialis anterior muscle, while considering the influence of maturation and sexual dimorphism.

Altogether, our results suggest that the tibialis anterior transcriptome is not shaped by sex, maturation, and diet as independent or merely additive variables. Instead, sex establishes an early transcriptional architecture, maturation represents the dominant remodeling axis, and HFHS exposure is associated with selective transcriptional responses that vary across sex-stratified analyses and exposure durations. Given the exploratory design, these results are best interpreted as transcriptional signatures that nominate biological processes for further validation rather than as direct evidence of functional remodeling.

Sex-dependent transcriptional organization emerged as an early and persistent feature of the tibialis anterior muscle in our study. This temporal window is particularly relevant because 7 and 23 weeks capture the transition from early post-weaning/juvenile muscle to young adult skeletal muscle, a phase during which sex-dependent transcriptional organization may still be consolidating. Recent work has characterized postnatal muscle maturation through approximately 7 weeks, often in male mice [25], whereas many obesity- and aging-related transcriptomic studies focus on adult or older animals [26,27,28]. Similarly, human transcriptomic studies have provided strong evidence for sex differences and sex-specific aging trajectories in adult skeletal muscle [29,30,31], but they do not address early postnatal maturation. Therefore, our 7- and 23-week comparison adds a less-explored developmental window, showing that sex-specific transcriptional dimorphism is already detectable near early maturation and becomes reinforced by young adulthood, before the onset of classical aging.

Although the analysis of the transcriptomic profile from peri-pubertal to adulthood stages was not our main focus, maturation emerged as the strongest organizing factor of the tibialis anterior transcriptome in our study, producing broader transcriptional separation than the HFHS diet alone. This is consistent with substantial age-related remodeling of skeletal muscle gene expression across rodent muscles [26] and in human muscle [32]. However, the predominance of maturation should not be interpreted as evidence that diet-induced changes are biologically minor. Instead, our data indicate that HFHS exposure acts within a strong maturation-dependent background, generating more restricted but clearly organized transcriptional responses whose patterns differed across the sex-stratified analyses and exposure duration. This interpretation is supported by recent mouse studies showing that obesity and aging can produce partly distinct, and sometimes interacting, effects on skeletal muscle gene expression and mitochondrial/metabolic function [22,28]. Similarly, transcriptomic analyses comparing fast/glycolytic and slow/oxidative muscles indicate that aging and obesity remodel skeletal muscle in a muscle-type-dependent manner, which is particularly relevant for tibialis anterior as a predominantly fast/glycolytic muscle [33]. Therefore, our findings suggest that maturation defines the main transcriptional landscape of tibialis anterior muscle, whereas the HFHS diet imposes selective diet-responsive modules that are not simply proportional to the number of differentially expressed genes, but instead reflect biologically meaningful metabolic, structural, extracellular matrix, and regulatory adaptations.

The sex-stratified analyses also revealed different transcriptional patterns following HFHS exposure at the two examined time points. In females, HFHS induced a stronger early response at 7 weeks, dominated by ECM- and tissue-organization processes, whereas the response at 23 weeks was more restricted and enriched for regulatory programs, including circadian and transcription-related ontological terms. Males showed the opposite pattern in DEG numbers, with a limited early response to HFHS, but prolonged exposure produced broader transcriptional remodeling involving lipid metabolism, contractile and structural processes, ECM-related terms, and signaling. These findings are consistent with previous mouse studies showing that high-fat or high-fat (HFD)/high-sugar diets affect skeletal muscle in a sex-dependent manner [22] and that long-term HFD exposure induces cellular and molecular changes that differ between male and female skeletal muscle [34]. More broadly, the sex-dependent response to an obesogenic diet is also supported by evidence that genetic background and sex strongly influence the metabolic outcome of high-fat feeding in mice [35]. Our study extends these observations by adding a temporal transcriptomic dimension, suggesting that the female tibialis anterior muscle responds rapidly to HFHS exposure through early matrix and tissue-remodeling programs, whereas male muscle develops a delayed but more extensive metabolic and structural response after prolonged exposure.

Extracellular matrix-related pathways appeared repeatedly across our analyses, particularly in the female early HFHS response and in selected age–diet intersections. This recurrence suggests that the ECM may represent one of the main transcriptional interfaces between maturation and HFHS exposure. In skeletal muscle, the ECM is not only a passive structural scaffold, but an active component of the muscle microenvironment that influences mechanical properties, cell signaling, vascular organization, regeneration, and metabolic exchange. Previous studies have shown that aging is associated with extensive remodeling of the skeletal muscle extracellular matrix and that these changes can contribute to impaired muscle stem cell function and altered tissue homeostasis [36]. Single-cell studies have also demonstrated that skeletal muscle contains diverse resident stromal, endothelial, immune, and progenitor cell populations that may contribute substantially to matrix-related transcriptional signals detected in bulk RNA-seq [37]. Moreover, recent transcriptomic analyses of tibialis anterior regeneration across the lifespan showed that age modifies the state of muscle stem and niche-associated cell populations [27]. The ECM results should therefore be viewed as a matrix-associated transcriptional signature, not as evidence of fibrogenesis. Nevertheless, the repeated appearance of ECM-related terms indicates that the muscle microenvironment may represent an early HFHS-associated transcriptional feature, particularly in the female comparison.

The male response to the HFHS diet was characterized by a delayed but broader transcriptional expansion, particularly after prolonged exposure. At 23 weeks, male HFHS muscle showed enrichment of pathways related to fatty acid metabolism, carbohydrate handling, triglyceride metabolism, contractile structure, extracellular organization, and signaling. This pattern suggests that in male tibialis anterior muscle, the transcriptional consequences of HFHS exposure are buffered or delayed, but later develop a more extensive metabolic and structural rewiring. These data are consistent with studies showing that HFD-induced obesity affects skeletal muscle gene expression, mitochondrial function, and substrate metabolism in an age-dependent manner [28] and remodels lipid-related networks [38]. In addition, comparisons between fast/glycolytic and slow/oxidative muscles indicate that aging and obesity can affect muscles differently according to their intrinsic metabolic and fiber-type properties [33]. Since tibialis anterior is predominantly fast/glycolytic, the late male enrichment for metabolic and contractile pathways may reflect a specific vulnerability or adaptive response of this muscle type to prolonged obesogenic stress. More importantly, this interpretation is coherent with our previous analysis in the same mouse cohort, where we showed that male soleus muscles accumulate intramyocellular lipids and exhibit a decrease in MyHC I fibers, accompanied by an increase in MyHC I/II hybrid and MyHC II fibers [22]. Thus, our data suggest that male HFHS exposure is not transcriptionally silent at early stages, but its broader consequences become more evident after sustained dietary challenge.

The smaller 23-week female DEG set under the HFHS diet included circadian and transcriptional regulatory terms. High-fat feeding can perturb peripheral clock-related gene expression and metabolic rhythmicity [39], and feeding time can influence the skeletal muscle clock, insulin sensitivity, and muscle transcriptome [40]. Circadian systems are also closely linked to glucose and lipid metabolism [41]. However, all tissues in the present study were collected at a single time point, so the observed enrichment cannot distinguish altered rhythmicity, phase, or a non-rhythmic change in clock-associated genes. We therefore regard this as a hypothesis-generating clock-associated signature rather than evidence of circadian disruption.

The intersection analyses further showed that overlap between maturation and HFHS responses was modest and differed between the female and male analyses. In females, the largest shared set linked maturation with the 4-week HFHS response and was enriched for ECM-related terms; in males, maturation shared oxidative/lipid metabolic terms with the 4-week response and a larger set of tissue-organization and FGFR-related terms with the 20-week response to diet. These overlaps identify transcriptional modules that may be sensitive to both developmental stage and diet, but they do not demonstrate that HFHS recapitulates maturation-associated biology.

At the individual-gene level, the small intersections highlighted candidates that may connect the pathway-level findings with known muscle biology. Immunoglobulin-Like and Fibronectin Type III Domain-Containing Protein 1 (Igfn1), shared across all female comparisons, encodes the Z-disk-associated protein implicated in myoblast fusion and differentiation [42,43]. Interestingly, skeletal muscle from males presented a reduction in Troponin C1 (Tnnc1), a calcium-binding protein expressed in slow skeletal muscle fibers and the heart, which can be attributed to a fiber-type transition from slow- to fast-twitch fibers, as previously observed in this model [22].

We also found several transcripts involved in metabolic pathways. Both female and male muscles presented the up-regulation of the sterol regulatory element-binding transcription factor 1 (Srebf1) that binds to the sterol regulatory element-1 (SRE1), which is a motif present in the promoter of the LDL receptor gene and other genes involved in sterol biosynthesis [44]. This protein has been reported to be up-regulated in diabetic mice [45], and its overexpression has been shown to induce muscle atrophy [46]. Fatty acid-binding protein 3 (Fabp3), up-regulated after both 4 and 20 W, is a small cytoplasmic protein crucial for transporting long-chain fatty acids from the cell membrane to mitochondria for energy production, which has been suggested as a biomarker of muscle [47]. This evidence is in line with our previous data showing that the HFHS diet induces the accumulation of lipids and the increase in mitochondrial activity in male skeletal muscle [22]. From the same intersection analyses, we could observe that the Spinster Homolog 2 (Spns2) transcript is up-regulated after 4 W of the HFHS diet and down-regulated after 20 W. Spns2 is a crucial plasma membrane exporter for sphingosine-1-phosphate (S1P), which interacts with specific S1P receptors, triggering downstream pathways that are essential for myoblast differentiation into mature myotubes [48] and to protect muscle from damage [49]. Fructose-Bisphosphatase 2 (Fbp2), up-regulated in male mice fed a 4 and 20 W HFHS diet, is a gluconeogenesis regulatory enzyme that reduces glycolytic flux and promotes the gluconeogenic direction [50], is involved in thermogenesis [51], and associates with mitochondria during energetic stress [52]. Possibly, Fbp2 up-regulation is both attributable to both a response to an obesogenic environment created by the high sugar content of the HFHS diet, which induces muscle cells to convert excess glucose into glycogen, and to the search for a metabolic equilibrium. Intersection analyses also revealed the alteration of Zbed6, a transcription factor that works as a transcriptional repressor of insulin-like growth factor 2 (IGF2) transcription, regulating the growth of skeletal muscle and multiple organs [53,54]. Analogously to other transcripts, Zbed6 is up-regulated after 4 W of the HFHS diet and is stable after 20 W, leading to suggest that 4 W of HFHS induces compensatory mechanisms also at the level of IGF2 signaling, which are successively lost in longer HFHS diet regimens.

More broadly, large-scale mouse phenotyping and single-cell atlases show that skeletal muscle and its resident cell populations change across the life course in sex- and context-dependent ways [55,56]. Our findings fit this general framework by placing HFHS-associated gene expression differences within a strong developmental and sex-associated background. Because the animals were juvenile at dietary initiation and young adults at the later endpoint, the present study addresses maturation rather than biological aging and should not be interpreted as a model of sarcopenia or sarcopenic obesity.

Overall, the data support a model in which HFHS-associated transcriptional signatures are context-dependent rather than uniform. Female and male patterns differed at both endpoints, and maturation-associated DEG sets overlapped only partly with HFHS-responsive sets. The strongest inference supported by the present study is therefore about transcriptional organization and functional enrichment, not about causal mechanisms or functional impairment.

## 5. Limitations

Some limitations should be considered when interpreting the reported findings. First, our study is based on bulk RNA-seq of whole tibialis anterior muscle, which captures the integrated transcriptional response of myofibers and non-myofiber populations but cannot identify the precise cellular origin of each signal. This is particularly relevant for extracellular matrix-, vascular-, immune-, and stromal-related terms. Although with some limitations due to muscle fiber cell size and cell relative abundance, single-cell or spatial transcriptomic analyses could help determine whether HFHS-sensitive programs influence myofibers, fibro-adipogenic progenitors, endothelial cells, immune cells, or other resident cell populations.

Second, some interpretations are constrained by the experimental and statistical design. Although circadian-related enrichment was observed in late female HFHS muscle, our experimental design was not structured to assess circadian rhythmicity; time-course sampling would be required to determine whether HFHS alters muscle clock function. In addition, dietary responses were evaluated using sex- and age-stratified pairwise comparisons rather than formal sex-by-diet interaction models. Therefore, differences observed between female and male comparisons or between exposure durations should be interpreted as differences in transcriptional response patterns rather than definitive evidence of sex-by-diet or time-dependent interaction effects.

Third, transcriptomic changes do not necessarily imply corresponding changes in protein abundance, tissue architecture, or muscle function. However, some of the findings reported here are consistent with those of our previous study [22], in which we performed a comprehensive characterization of skeletal muscle, including capillary density, fiber-type composition, mitochondrial function, and lipid and glycogen content. These complementary morphological and metabolic data provide an important framework for interpreting the molecular signatures identified in the present study. Nevertheless, whether the transcriptional changes identified here translate into alterations at the protein and functional levels remains to be established. Since such validation was beyond the scope of the present discovery-based transcriptomic study, our findings should be considered hypothesis-generating. Finally, an additional limitation of this study could be represented by the relatively small number of biological replicates of each experimental group, for which no formal a priori power or sample-size calculation was performed. Nevertheless, the use of three to four biological replicates per group is consistent with previous exploratory RNA-sequencing studies investigating skeletal muscle responses to obesogenic diets. Since the limited sample size may reduce the statistical power to detect subtle transcriptional differences, the present findings should be considered exploratory and hypothesis-generating and will require further validation in larger cohorts.

## 6. Conclusions

In conclusion, this study suggests that the tibialis anterior muscle transcriptome is shaped by sex and maturation, while the HFHS diet is associated with distinct transcriptional patterns at the two examined exposure durations. The main contribution of our exploratory work is not only the identification of diet-responsive genes, but also the introduction of the hypothesis that diet responsiveness depends on the biological context in which it occurs. Our data suggest that sex-specific dimorphism is already present early, maturation represents the dominant transcriptional axis, and HFHS exposure engages selective modules that only partially converge with maturation-associated remodeling. Although the number of biological replicates is limited in this experimental work, these findings reinforce the importance of incorporating sex and developmental stage into studies of diet-induced skeletal muscle remodeling and suggest that early-life obesogenic exposure may shape muscle biology through distinct female and male transcriptional trajectories.

## Data Availability

The original contributions presented in the study are included in the article and in the Supplementary Material; further inquiries can be directed to the corresponding authors.

## Supplementary Materials

The following supporting information can be downloaded at: https://www.mdpi.com/article/10.3390/nu18203354/s1, Table S1: Comparison of gene expression profiles between female and male mice at 7 and 23 weeks; Table S2: Enrichment results at 7 weeks of age in female vs. male transcriptome; Table S3: Enrichment results at 23 weeks of age in female vs. male transcriptome; Table S4: Transcriptomic response to 4- and 20-week HFHS diet in females; Table S5: Enrichment results for 4-week HFHS diet in females vs. controls; Table S6: Enrichment results for 20-week HFHS diet in females vs. controls; Table S7: Transcriptomic response to 4- and 20-week HFHS diet in males; Table S8: Enrichment results to 4-week HFHS diet in males vs. controls; Table S9: Enrichment results to 20-week HFHS diet in males vs. controls; Table S10: Integrated enrichment summary data.
